# Approaches to genetic tool development for rapid domestication of non-model microorganisms

**DOI:** 10.1186/s13068-020-01872-z

**Published:** 2021-01-25

**Authors:** Lauren A. Riley, Adam M. Guss

**Affiliations:** 1 Biosciences Division, Oak Ridge National Laboratory, Oak Ridge, TN 37831 USA; 2 Bredesen Center, University of Tennessee, Knoxville, TN 37996 USA

**Keywords:** Non-model microbes, Genetics, Genetic tools, Transformation, Metabolic engineering, Synthetic biology

## Abstract

Non-model microorganisms often possess complex phenotypes that could be important for the future of biofuel and chemical production. They have received significant interest the last several years, but advancement is still slow due to the lack of a robust genetic toolbox in most organisms. Typically, “domestication” of a new non-model microorganism has been done on an ad hoc basis, and historically, it can take years to develop transformation and basic genetic tools. Here, we review the barriers and solutions to rapid development of genetic transformation tools in new hosts, with a major focus on Restriction-Modification systems, which are a well-known and significant barrier to efficient transformation. We further explore the tools and approaches used for efficient gene deletion, DNA insertion, and heterologous gene expression. Finally, more advanced and high-throughput tools are now being developed in diverse non-model microbes, paving the way for rapid and multiplexed genome engineering for biotechnology.

## Introduction

The world’s energy and chemical demand is ever-increasing, and currently, the demand for fuels and chemicals is primarily met with fossil fuels. Fossil fuels like petroleum not only provide the raw materials to make liquid fuel for the transportation sector, they also provide the building blocks needed for a wide variety of heavily utilized chemicals and materials, including plastics, perfumes, paints, fertilizer, and detergents [1]. However, fossil-derived carbon is inherently unsustainable, and a promising alternative is microbial conversion of renewable feedstocks. Microorganisms often have the ability to make drop-in or functional replacements for the fuels and chemical building blocks used today, but metabolic engineering is typically required to reach the titers, rates, and yields needed for a commercial process as well as to diversify the chemicals that can be produced.

Currently, model organisms like *Saccharomyces cerevisiae* and *E. coli* are most commonly used for metabolic engineering research and are often used for industrial biochemical production. Model organisms are attractive because they are industrially robust, decades of research have enabled deep understanding of the organisms, and they have a large genetic toolbox to enable rapid and simple modifications. However, engineering of synthetic pathways into these organisms is often most successful with short heterologous pathways, while long synthetic and heterologous pathways are generally not as robust and can cause a large metabolic burden [2]. To overcome the challenges with current industrial biochemical production and to make next-generation fuels and chemicals profitable, recent research has focused on non-model organisms that natively have phenotypes of interest for each unique feedstock and processing method [3].

Industrial bioproduction of fuels and chemicals initially focused on first generation feedstocks such as starch and sucrose. These feedstocks are easy to breakdown and convert, but potential competition with food crops, limited greenhouse gas emission benefits, and environmental sustainability concerns makes these feedstocks problematic [4]. Alternate feedstocks such as the sugars and aromatics in lignocellulosic biomass [5], sunlight or renewable electricity coupled with CO_2_ fixation [6, 7], syngas [8], and waste plastic [9] have the potential to be cheaper. These feedstocks are also more environmentally friendly sources of carbon for fuel and chemical production, but bioconversion of these substrates is much more challenging. However, many non-model microbes often have evolved to utilize these feedstocks efficiently, making them potentially attractive platforms for bioconversion. Unfortunately, many of the organisms with these native capabilities have poorly characterized metabolisms and are challenging to modify genetically, hindering metabolic engineering efforts.

Other traits potentially beneficial to industrial processing methods include tolerance to low pH, tolerance to high salt, or the ability to grow at high temperatures. These traits could streamline chemical production by reducing contamination, reducing the need for pH control and buffering, and increasing enzymatic reaction speed. These abilities are multigene, complex phenotypes that are difficult or impossible to engineer into model organisms. The more widespread use of non-model organisms could leverage these phenotypes to usher in a new era of biotechnology for biofuel and chemical production.

Two major barriers to the use of non-model organisms for metabolic engineering include a lack of genetic tools and limited knowledge of the organism’s physiology. Traditionally, physiological characterization of a new organism was time-consuming and labor-intensive, with much of the new knowledge generated through biochemistry and characterization of substrate utilization and product formation. It was difficult to gain additional in-depth physiological information to uncover cell metabolism and regulation [2, 10]. However, genomic knowledge has become easier to acquire due to the rise of next-generation sequencing techniques and the dramatic drop in the cost of genome sequencing [11, 12]. Using basic genomic knowledge, the groundwork can quickly be laid for systems-level biological analyses, or “omics” in non-model microbes. Enabling tools like transcriptomics and proteomics are straightforward to apply to new microorganisms, and they assist in addressing fundamental questions and provide measurements for all cellular components [10]. The cost and time associated with these tools are also dropping, and the major challenge is now turning these large datasets into usable knowledge. Integration of multi-omics data to models helps give a more comprehensive dataset and helps to link the genotypes associated with the complex phenotypes of interest [13, 14]. Having comprehensive knowledge of a microorganisms’ metabolic pathways and flux is important to successful metabolic engineering [15]. With the increased availability of affordable -omics analyses and DNA synthesis, the major remaining barrier to widespread adoption and “domestication” of non-model microbes is the lack of robust genetic tools.

## Barriers to genetic modification and enabling transformation

In order to develop non-model organisms for bioprocessing, genetic modification is typically needed. The development of genetic tools requires the ability to efficiently transform DNA into a target organism. There are four primary barriers to overcome for successful transformation of bacteria: cellular uptake of foreign DNA, evasion of native immune systems that degrade foreign DNA, selection for transformants, and stable maintenance of foreign DNA by the microbial host. Each poses a challenge, and the largest barrier is often the evasion of host defense systems.

### DNA entry into the cell

The first challenge, introducing DNA into cells, requires DNA to get past one or two membranes, as well as other physical barriers, such as peptidoglycan, to reach the cytoplasm. Depending on the organism, a variety of techniques can be utilized, including commonly used methods such as electroporation, conjugation, protoplast transformation, and natural competence. For electroporation, an electrical field is applied to a mixture of DNA and washed cells, which opens holes in the membrane, and enables the uptake of DNA [16, 17]. Electroporation works broadly across phylogenetic groups [18–23], including in diverse bacteria, eukaryotes, and archaea, and therefore is often the first method attempted by researchers trying to transform a new organism. Conjugation enables DNA transfer through direct cell to cell contact from a donor cell to the recipient. This technique has been used in diverse organisms using a donor *E. coli* strain carrying broad host range conjugation machinery [24–26]. DNA is transported into the cell as single stranded DNA (ssDNA), which is usually highly recombinogenic, making it an especially good method of transformation when homologous recombination is desired [27–29]. Conjugation can be more challenging into microorganisms that have a vastly different optimal growth conditions than *E. coli*, such as halophiles or thermophilic anaerobes. In protoplast transformation, the bacterial cell wall is degraded through the introduction of lysozyme. Removal of the cell wall allows for cells to uptake DNA without a barrier. This is a technique that has seen wide use the actinomycetes [30–33]. Natural competence, on the other hand, is a mechanism driven by suite of genes that allows some bacteria to uptake DNA from their environment [34], and this imported DNA is also single stranded. Transformation using this technique is often relatively simple in strains containing the natural competence genes, where the strain of interest is incubated with DNA under conditions where the competence genes are expressed. Prominent examples of hosts that use natural competence for transformation include the Firmicute *Bacillus subtilis* and the Gammaproteobacterium *Acinetobacter baylyi* [35, 36]. In strains that are not naturally competent, conjugation and electroporation are the most common methods of transformation, with electroporation often enabling the highest transformation efficiencies [37]. Though these three techniques are the most commonly used, there are many less commonly used techniques reported in the literature, including phage-enabled transfer [38], chemically induced competence [39], sonoporation [40], biolistic bombardment [41], liposome-mediated fusion [42], and nanofiber piercing [43]. Because these methods are not used as widely, their application to new organisms is relatively underexplored.

### Host defense systems

Prokaryotic organisms have evolved multiple defenses against foreign DNA, which can present a major barrier to DNA transformation. Restriction-Modification (RM) systems act as innate immune systems and are one of the primary systems used by prokaryotes to protect themselves against foreign DNA. Cells recognize and degrade DNA that is methylated differentially from that of its own DNA [44]. Almost 90% of prokaryotes encode RM systems, with most encoding two or more [45]. Microbes often also encode adaptive immune systems, including clustered regularly interspaced short palindromic repeats (CRISPR). CRISPR systems natively protect prokaryotes against foreign DNA, for instance from bacteriophage. Viral derived DNA sequences are acquired during an infection and inserted into the chromosome where they may be transcribed to prevent against repetitive infection [46]. Native CRISPR systems are unlikely to pose a major barrier to DNA transformation in most cases because the specific sequences acquired by the host CRISPR system are unlikely to be present in the DNA that is being transformed. Therefore, CRISPR will only be further discussed in this review in relation to their use as genetic tools. Several other defense systems have been discovered, but they are rare and/or poorly characterized. These include abortive infection (Abi), BREX, Dnd, Dpd, DISARM, pAgos, and others [47, 48]. The impact of these systems on genetic transformation is currently unknown, but not yet observed to present major barriers.

To enable genetic modification, transformed DNA must evade the native immune systems of the host. RM systems are the most important immune system that DNA needs to evade, and many studies have shown the importance of this for successful transformation (e.g., 49–58). Of the four classes of RM systems, Types I, II, and III typically comprised two primary components, a restriction enzyme and a methyltransferase. The methyltransferase modifies a specific base within a specific motif throughout the host chromosome so that the genome is protected from restriction enzyme cleavage, with modifications including 6-methyladenine (m^6^A), 4-methylcytosine (m^4^C), or 5-methylcytosine (m^5^C). The restriction enzyme cleaves the same motif in unmethylated DNA that enters the cell. Type IV systems, on the other hand, only consist of a nuclease that cleaves methylated DNA, with the recognized motifs different than those targeted by native methyltransferases from Types I, II, and III. The sequence specificity of Type IV systems is typically poorly characterized and is a ripe area for future research.

To evade RM systems, the first step is to identify the methylated motifs within a cell. Microbial methylation sites are most commonly determined through genome sequencing with single molecule real-time (SMRT) sequencing on the PacBio platform [59, 60]. SMRT sequencing routinely identifies m^6^A and m^4^C motifs and, with a significantly lower efficiency, m^5^C motifs through kinetic delays in nucleotide incorporation at modified bases during sequencing. While eukaryotic methylome analysis often employs whole genome bisulfite sequencing (WGBS) to detect m^5^C motifs, it has rarely been used in bacteria. However, robust detection of m^5^C is critical for complete methylome analysis [50]. Other emerging techniques, such as nanopore sequencing, may also be used for methylome analysis [61, 62]. New England Biolabs (NEB) has created a database, REBASE, of all known RM systems (both experimentally determined and computationally predicted) and publicly available microbial methylomes to help in the identification of RM systems [63]. Not all methyltransferases are associated with restriction enzymes; they can also play a regulatory role in DNA replication, gene expression, and DNA mismatch repair [64]. However, the methylated motifs reveal the maximum number of sites that could be subject to restriction in a given host.

Once the methylated motifs are identified, several methods have been used to evade the corresponding RM systems. One approach is transforming DNA that lacks the identified motifs, or mutating DNA so that it no longer contains the motif [50, 65, 66]. Recently, software has been designed to aid in the process of eliminating restriction sites [67]. This can often be done for uncommon motifs, but it cannot always be used for shorter, more common motifs, like four base pair recognition sequences that may exist in plasmid origins of replication or other DNAs that require a specific sequence, such as those needed for homologous recombination.

Another way to overcome RM systems is by methylating DNA of interest in the same way as the target host prior to transformation, such that the organism does not recognize it as foreign. The most common approach to achieving methylation is to express the target organism’s restriction-associated methyltransferases in *E. coli*, and then isolate the DNA of interest from this *E. coli* strain to properly methylate it prior to transformation. Early pioneering work demonstrated transformation of *Clostridium acetobutylicum* by first methylating plasmid DNA in *E. coli* with the methyltransferase from *B. subtilis* phage phi3T [49]. This approach was expanded with a method called plasmid artificial modification (PAM), which introduced all the methyltransferases from a given strain into *E. coli* using plasmid-based expression, followed by isolation of the DNA of interest out of the PAM host to properly methylate it prior to transformation of the target organism [68]. More recently, informed by methylome analysis, only those methyltransferases identified as important were introduced to the *E. coli* chromosome to more stably express the enzymes and to only mimic the methylome data.

DNA can also be properly methylated through in vitro methylation [69]. Some enzymes are commercially available, which can be especially useful for 4-base motifs that are targeted by commercially available methyltransferases. For those motifs that are not methylated by commercially available enzymes, cell-free extracts can be used, as demonstrated in *Helicobacter pylori* [70] and *Saccharopolyspora spinosa* [71], to increase transformation efficiency.

RM systems can also be partially evaded by the transformation mechanism chosen. Often, electroporation is unsuccessful when using improperly methylated DNA and, in these cases, using conjugation can yield successful transformation. The conjugation machinery transfers single stranded DNA (ssDNA) that can avoid restriction enzyme cleavage until it has time to become properly methylated [72]. Similarly, natural competence also imports ssDNA and can reduce the impact of restriction systems [73].

Another important consideration when transforming a new bacterial strain is the presence of Type IV restriction systems, which degrade methylated DNA motifs. Researchers typically isolate plasmid DNA from *E. coli* prior to transformation into their desired host, and commonly used laboratory *E. coli* strains encode two major DNA methylases, *dam* and *dcm*, targeting G(m^6^A)TC and C(m^5^C)WGG, respectively. Many potential target strains encode *dam*, either as part of an RM system or as a housekeeping methyltransferase, and a few encode *dcm*, but many do not. Therefore, it is important to isolate plasmid DNA from an appropriate *E. coli* genetic background, using strains deleted for *dam* and/or *dcm* when the target organism does not methylate the same site. This prevents unnecessary DNA methylation and subsequent degradation by Type IV systems, which has been demonstrated in many strains [74–76]. It is also important to utilize an *E. coli* strain that lacks the native Type IV systems (*mcrA*, *mcrBC*, and *mrr*) when expressing methyltransferases. Methylation of motifs recognized by the native type IV systems would cause *E. coli* to restrict its own chromosome, which would kill the *E. coli* strain.

RM systems are frequently acquired via horizontal gene transfer [48], and therefore methods to improve transformation in one strain typically do not directly translate to closely related strains. These systems tend to be hyper-variable within an environmental community of closely related strains, such that if a phage happens to avoid one cell’s restriction systems and becomes methylated like that host, it does not eliminate the entire population [45]. Therefore, methylome analysis and RM system evasion must be developed for each desired host, even if they are strains of the same species.

### Maintaining and selecting for DNA

Once DNA has entered the cell and evaded degradation, it needs to be maintained during cell division, and transformed cells must be selected from the untransformed cells that constitute the majority of the population. DNA can be maintained either through autonomous replication or through chromosomal integration. Plasmid-based autonomous replication is typically the best way to achieve the highest transformation efficiency for a new microbe and is the most commonly used method to demonstrate initial transformation. For metabolic engineering, however, plasmid-based gene expression has drawbacks, such as high copy number artifacts, variable plasmid copy number, the need for continuous antibiotic selection, and a high metabolic burden. Therefore, chromosomal integration is also an important tool, and different approaches to developing DNA integration genetic tools will be discussed in more detail below.

Development of a plasmid-based DNA maintenance system relies on identification of an origin of replication that functions in the target organism. The most common vectors used for cloning and replication in *E. coli* use origins such as pUC, ColE1, and p15a, each with varying copy numbers [77], but often these origins do not replicate in non-model bacteria. Therefore, origins that function in diverse bacteria are needed. Broad host range plasmids are capable of transfer and maintenance in bacteria from different phylogenetic subgroups, examples include pBBR1, pRK2, pBC1, and many others [78]. Plasmid libraries have been formed around the broad host-range origins of replication and used in a variety of organisms [79]. Alternatively, origins can come from native plasmids of the strain or close relatives [80, 81]. If none are present, replicating plasmids may be constructed by cloning the chromosomal origin of replication to create a mini-chromosome [82, 83].

Next, one must select for cells that were transformed and eliminate cells that were not. The most common positive selectable markers are antibiotic resistance genes (Table 1). To determine the marker(s) most likely to work, the minimum inhibitory concentration (MIC) is determined by exposing the host bacteria to increasing levels of a panel of antibiotics. This is most easily done in liquid growth medium, but higher concentrations of antibiotic may be needed for selection on agar plates. Another consideration when choosing antibiotic resistance markers is compatibility with an organism’s growth conditions. For example, when working with thermophiles, one should focus on antibiotics that are more stable at higher temperatures like thiamphenicol and kanamycin [84] and thermotolerant selectable markers [85]. A less commonly used alternative to antibiotics is nutritional selection. In this case, an auxotrophic strain is generated by deletion of an essential nutrient gene and transformed with a plasmid encoding the missing biosynthesis gene(s). The resulting transformants are selected on media lacking the target nutrient [86, 87]. Libraries of modular plasmids combining antibiotic resistance markers, origins of replication, multiple cloning sites (MCS), and other genetic parts have been developed for different classes of organisms, and they are very useful for the rapid testing of genetic parts [88–90].

**Table 1:** Commonly used antibiotics and corresponding selectable markers

Antibiotic	Marker	Class
Kanamycin/neomycin	*neo*	Aminoglycoside
Chloramphenicol/thiamphenicol	*cat*	Chloramphenicol
Erythromycin	*ermB, ermF*	Macrolide
Ampicillin/carbenicillin	*bla*	Beta-lactam
Tetracycline	*tetA*	Tetracycline
Bleomycin	*ble*	Glycopeptide
Gentamicin	*aacC1*	Aminoglycoside
Streptomycin and spectinomycin	*aadA*	Aminoglycoside
Zeocin	*zeo*	Glycopeptide
Apramycin	*apr*	Aminoglycoside
Thiostrepton	*tsr*	Cyclic oligopeptide
Puromycin	*pac*	Aminonucleoside
Hygromycin	*hph*	Aminoglycoside
Nourseothricin	*nat*	Aminoglycoside

Native antibiotic resistance levels can vary widely between strains. Therefore, an MIC experiment should be performed with each target organism to determine the level of antibiotic needed for selection.

## Chromosome modification tools in non-model microorganisms

After demonstration of initial transformation, more advanced tools can be developed to enable efficient genome editing and ultimately high-throughput strain engineering. Genome editing can allow for gene deletions, insertion of heterologous pathways, point mutations, and altered gene regulation. Enabling genome integration and deletion tools facilitates rational metabolic engineering, where competing production pathways can be eliminated, and new pathways can be stably introduced. Development of more sophisticated tools is critical to the engineering of non-model organisms for high rates and titers of desired products.

### Homologous recombination

**Fig. 1:**
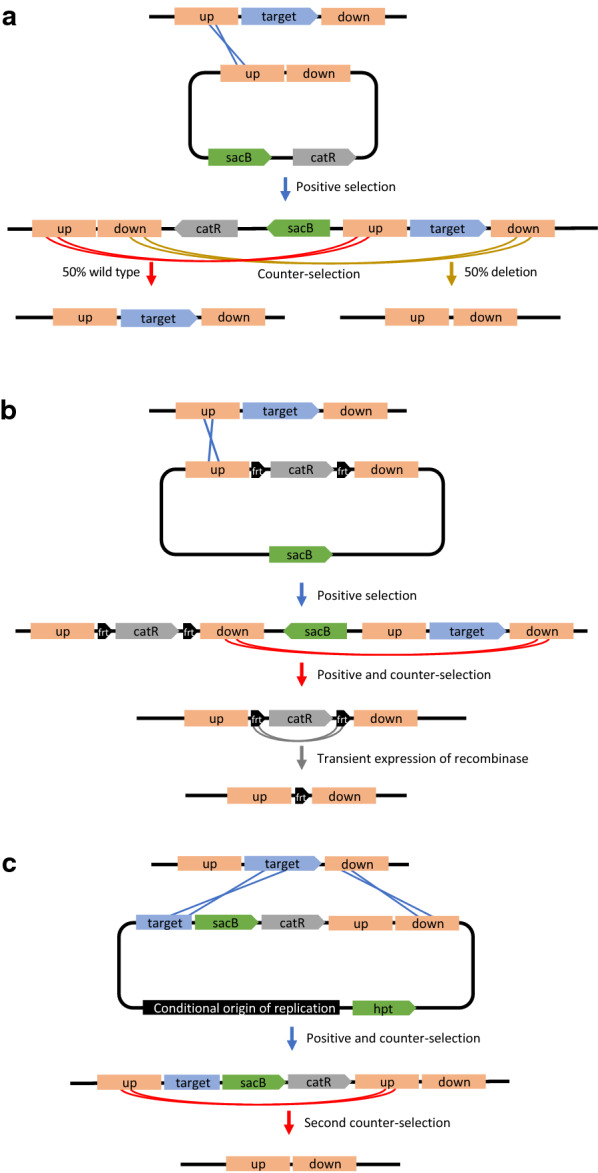
Methods using homologous recombination for gene deletions, with details for each method described in the text. **a** Basic homologous recombination using a non-replicating plasmid where 50% of colonies will be the desired deletion. **b** Homologous recombination using the positive selectable marker between the homology arms, flanked with recombination sites, and **c** homologous recombination using a replicating vector and three homology regions. Orange, homology regions, with “up” representing the DNA region upstream of the target gene, and “down” representing the DNA region downstream of the target gene; blue, target gene for deletion; green, counter-selectable markers; gray, positive selectable marker; crossed lines, sites of recombination

Homologous recombination is a naturally occurring mechanism essential for DNA repair in bacteria, and it can be leveraged to rationally introduce modifications to an organism’s chromosome. The most commonly deployed method uses a non-replicating plasmid-based technique to create a scar-less mutation [91]. Typically, this is mediated by nuclease–helicase complex, RecBCD, and the single stranded DNA-binding DNA repair protein, RecA [92]. To use homologous recombination for gene deletion, a plasmid is required that contains a selectable maker, a counter-selectable marker, and DNA that is homologous to the upstream and downstream regions of the gene targeted for deletion (Fig. 1a). The lengths of homologous DNA regions are often around 500–1000 bp each. After transformation of the plasmid into the microbe, the plasmid recombines into the chromosome at one region of homology to integrate the entire plasmid, which is selected via the positive selectable marker contained on the plasmid. A second recombination event will resolve the merodiploid into either the parent chromosome or a gene deletion. If the first recombination event is repeated, the strain reverts to wild type, but if the recombination event occurs in the second region of homology, the targeted gene is deleted. When there is no fitness defect associated with the deletion, the frequency of deletion should be approximately 50%, which can be easily screened via PCR.

Selection for resolution of the merodiploid requires a counter-selectable marker, which allows selection for loss of DNA. Numerous counter-selections have been demonstrated (Table 2), including *sacB* in Gram-negative bacteria and *pyrF* and *hpt* in Gram-positive bacteria [91, 93–96]. Some of the markers are less desirable than others, though. Markers such as *sacB* often do not function well in Gram-positive bacteria [93]. There are also markers such as *pyrF* that can act as both a selectable and counter-selectable marker making them more flexible [97–99], but in many cases, they also result in auxotrophy, requiring growth on minimal medium for prototrophic selection of transformants. Many genes used as counter-selectable markers are natively encoded in the host genome. In these cases, the native copy must first be deleted before it can be used as a counter-selectable marker. However, the presence of a native counter-selectable marker also presents an opportunity for metabolic engineering, as it can serve as a simple site for insertion of heterologous DNA with a direct selection for gene replacement. Importantly, inactivating mutations (e.g., frameshifts, active site mutations, etc.) in counter-selectable markers have the same phenotype as recombinants and arise spontaneously, resulting in a background of cells that do not contain the targeted deletion. Therefore, counter-selections often require more screening to find correct clones relative to positive selection. In the absence of reliable counter-selectable markers, screens can be used, but this is much less efficient and should be avoided if possible [100].

**Table 2:** Commonly used counter-selectable markers and associated selections for homologous recombination in bacteria

Marker	Counterselection	Reference
*sacB*	Sucrose	[101]
*upp*	5-Fluorouracil	[102]
*hpt*	8-Azahypoxanthine, others	[103]
*tdk*	5-Fluorodeoxyuridine	[104]
*pyrF/ura3*^a^	5-Fluoroorotic acid	[105]
pheS*(A294G)	*p*-Chloro-phenylalanine	[106]
*codA*	5-Fluorocytosine	[107]
Inducible *mazF*	N/A; expression is toxic	[95]
*galK*	2-Deoxygalactose	[108]
*apt*	2-Fluoroadenine	[109]
*rpsL(strA)*	Streptomycin	[110]
*tetAR*^a^	Fusaric acid	[111]
*thyA*	Trimethoprim	[112]
Inducible *ccdB*	Expression is toxic	[113]
*oroP*	5-Fluoroorotate	[114]
*pta-ack*	Chloroacetate	[115]

^**a**^Can be used as a positive selectable marker with uracil prototrophy and tetracycline resistance, respectively

Another approach is to place the antibiotic resistance marker between the homology arms, allowing for direct selection of the gene deletion (Fig. 1b) [116]. This approach is especially useful when the mutant phenotype is deleterious, which would make the approach in Fig. 1a challenging. This approach can also work in the absence of a counter-selectable marker when using conjugation or natural competence and selecting directly for the double-recombination event that replaces the gene target with the antibiotic resistance gene. Often the antibiotic resistance marker is flanked by recombination sites, like *frt* or *lox*, so that the marker can be removed after integration into the chromosome by introduction of the corresponding recombinase, like Flp or Cre [116–118].

The frequency of homologous recombination varies widely in bacteria and in some cases is a rare event, making the transformation efficiency of integrating plasmids multiple orders of magnitude lower than for replicating plasmids. Therefore, replicating plasmid-based homologous recombination methods can be an attractive approach when the combination of transformation efficiency and homologous recombination in the target organism is low. However, because the plasmid replicates autonomously, it can be challenging to select chromosomal modifications; therefore, alternate approaches are needed.

One option is to use a conditional origin of replication, such as a temperature-sensitive plasmid. This allows the transformation event and the homologous recombination event to be uncoupled, where the plasmid can be transformed at a permissive temperature, generating a whole population of cells that contain the plasmid. Upon moving the culture to the non-permissive temperature, one can select for the cells that have performed recombination. Selection then can proceed as in Fig. 1a.

Alternatively, introduction of a third region of homology and a second counter-selectable marker can allow for selection of each recombination event and plasmid loss (Fig. 1c) [20]. In this approach, the replicating plasmid is first transformed into the strain. Then, while maintaining selection for the antibiotic resistance gene, selection is performed against the counter-selectable marker that is on the plasmid backbone. This selects for two recombination events to insert the antibiotic resistance onto the chromosome and simultaneous plasmid backbone loss. The resulting merodiploid can then be resolved by selecting against the second counter-selectable marker, resulting in the desired deletion.

Homologous recombination based genetic tools have been used in a variety of non-model organisms to increase biochemical production and enable non-native carbon catabolism though the deletion of competing pathways and insertion of heterologous genes [119–122].

### Recombineering

Methods to increase the efficiency of homologous recombination have been developed, often called recombination-mediated genetic engineering, or recombineering [123, 124]. By increasing the recombination efficiency, construction of gene deletions, insertions, and point mutations is more efficient. Recombineering uses proteins like those derived from the lambda bacteriophage Red complex, Beta, Exo, and Gam, to integrate single stranded and linear DNA into the genome, often using short homology arms to guide recombination. Recombineering is widely used in *E. coli*, and the genes associated with lambda Red have also been demonstrated to function in other proteobacteria, like *Pseudomonas putida* [125]. Recombineering can be difficult to develop in non-model organisms because lambda Red genes are often not functional in those hosts. Considerable effort has gone towards identifying genes analogous to lambda Red and the recombination protein RecT in phylogenetically diverse organisms [126, 127]. Using this approach, recombineering has been demonstrated in a variety of other strains, including *Clostridium acetobutylicum* [128], *Lactococcus lactis* [129], *Clostridium thermocellum* [130], and others [126, 131, 132]. The recombinases can then be used to enable ssDNA recombination for a recombination techniques often requires 300–1000 bp of homology for efficient recombination, while recombineering techniques can shorten this to as few as 20 bp.

### Random DNA insertion

Non-homology-based approaches are also useful when engineering non-model microbes. One such approach is transposition. Transposons allow random integration of DNA segments throughout the genome with no required homology. Therefore, transposition is traditionally used to create a library of insertional disruptions that can be screened for particular phenotypes. These phenotypes can then be correlated with genotypes by identifying the site of insertion [133]. Commonly used transposons include Tn5, which inserts mostly randomly but is known to favor “hotspots”, and *Himar1*, which can insert randomly between any TA dinucleotide [134, 135]. Recently, random transposon mutagenesis has been combined with high-throughput sequencing for genome-scale analyses, called TnSeq [136]. Further combination with randomly barcoded transposons simplifies reverse genetics, where a pooled library of barcoded insertional mutants can be created, and the bar codes can be mapped to the genome to identify disrupted genes [137]. A barcoded library can be an incredibly powerful tool for genome-scale analyses. A list of putatively essential genes can be compiled by identifying the genes that were not disrupted in the library. Furthermore, genes related to phenotypes such as solvent and organic acid resistance, product formation, and carbon assimilation can be identified by finding barcodes that increase or decrease in abundance under selective conditions [136, 138, 139].

Transposons can also be used to insert heterologous pathways into bacteria [140], which have been applied to non-model microbes such as *C. ljungdahlii* for acetone production [141] and *Acidothiobacillus ferroxidans* for isobutyric acid biosynthesis [142]. Transposons have also been combined with the Cre-*lox* site-specific recombination system to insert a landing pad, followed by site-specific insertion of heterologous pathways [143]. Transposons are especially useful for application in organisms that lack other genetic tools such as homologous recombination for integrating DNA into the chromosome. Integrating DNA randomly has its drawbacks, however. The transposon often integrates into coding regions of the genome, which can interfere with the fitness of the strain, and identifying the location of transposition requires additional effort. Random integration also does not enable comparison of a construct across genetic backgrounds because the transposition event occurs in different loci, which can result in differing levels of gene expression for the construct [144].

### Site-specific DNA integration

Site-specific recombinases have been a valuable tool for DNA insertions and excisions where the enzyme catalyzes a recombination event between two specific DNA sequences, sometimes called attachment (*att*) sites. The most commonly used site-specific recombinases are the Cre and Flp that enable a reversible recombination (integration or excision) reaction at identical sites, *loxP* and *frt*, respectively [145]. Both systems have been used to remove antibiotic resistance markers by integrating a cassette into the chromosome with *loxP* or *frt* flanking the marker followed by transient expression of the corresponding recombinase [123]. For example, Flp/*frt* is widely used in the *E. coli* lambda Red system to remove the antibiotic resistance marker, and it has also been used in a variety of non-model organisms, including thermophiles, methanotrophs, and biofuel producing *Clostridium* species [94, 116, 122, 146]. One drawback to using Cre and Flp, however, is that the *loxP* or *frt* scar left behind can cause genetic instability when multiple modifications are stacked into a single strain, leaving behind multiple, identical copies of the scar that can all serve as future substrates for the recombinase. Using mutated sites can help overcome the genome instability [147, 148].

A second group enables a site-specific recombination event at non-identical attachment sites, typically called *attB* (bacterial) and *attP* (phage), reflective of the fact that these recombinases often derive from bacteriophage that lysogenize their hosts. Recombination creates two new sites, *attL* (left) and *attR* (right). The integration event is unidirectional because the *attL* and *attR* are not substrates for recombination, so the recombinase cannot excise the construct from the genome [149]. Some unidirectional recombinases, including the lambda integrase, require host machinery to function, which limits use outside of their native host or very close relatives. Lambda phage also has relatively large attachment sites of 350–450 bp each. Alternatively, the large serine recombinases, as exemplified by the archetypical ΦC31 integrase, can function in a broad-host range because they do not require host machinery. This has led to their development as genetic tools in a wide variety of microbial hosts, especially in the actinomyces [150–153]. Due to their utility for rapid and high efficiency integration of DNA, they have been used to test genetic libraries at single copy on the chromosome, such as promoter libraries [150], and to integrate heterologous DNA [154–156]. Genetic tools developed with serine integrases can rely on native or pseudo-*att* sites that are present in the genome [157] or non-native *att* sites that have been previously integrated into the genome [154, 158, 159]. One of the most commonly used integrases is ΦC31, which was identified from *Streptomyces* and is commonly used in a DNA integration system in this genus [28, 153]. Multiple orthogonal recombinases have been identified and characterized [149, 153], setting the stage for them to be used much more broadly. Another type of site-specific recombinase includes transposases that target highly conserved sequences like Tn7 and Tn1545 [144]. Tn7 has been demonstrated to function in more than 20 bacterial species. It enables integration into the highly conserved *attTn7* site downstream of the *glmS* gene. This makes the use of site-specific transposons more desirable for some applications than the random transposons for integration of heterologous constructs [144].

### CRISPR/Cas

A recent and potentially universal tool for engineering non-model microbes uses clustered regularly interspaced palindromic repeats (CRISPR) and CRISPR-associated (Cas) proteins. Native CRISPR–Cas systems comprise a CRISPR array containing spacers separated by short repeats. Spacers become incorporated into the array when the microorganism encounters invading DNA to enable the microorganism to recognize and attack that same sequence when they encounter it again [160]. Many of the systems cleave DNA at this sequence using a Cas nuclease that is directed by a guide RNA (gRNA). Cas9 was first identified in *Streptococcus pyogenes* (spCas9), and it introduces a double-stranded break (DSB) at a specific sequence guided by a sgRNA adjacent to a protospacer adjacent motif (PAM) [161, 162]. Natively, the gRNA is derived from the CRISPR array, and in the case of the most utilized type II Cas nuclease, Cas9, the array is transcribed and processed (crRNA) with trans-acting RNA (tracrRNA) into a single guide RNA (sgRNA)[163]. In heterologous systems, the sgRNA can be transcribed as a single RNA, simplifying the number of genetic parts needed for engineering. If a template containing the desired modification is also included, the DSB can be repaired, resulting in an edited genome.

CRISPR–Cas systems have been leveraged in a wide variety of organisms to introduce scarless point mutations, insertions, and deletions in the genome. The most widely used CRISPR nuclease is spCas9 because, unlike many of the other Cas proteins, it is a single enzyme that only requires the introduction of a synthetic gRNA and the Cas9 nuclease to enable genome editing. CRISPR-spCas9 was first introduced as a genome engineering tool for bacteria in *E. coli* [162, 164] and has now been implemented in a wide variety of species using a plasmid for the repair template and an organism’s native homologous recombination machinery, as recently reviewed in [162]. Cas9-based editing has been further developed by combination with recombineering machinery to enhance the efficiency of DSB repair, and allowing use of a linear recombineering template with homology to the edited region [164, 165]. The combination of CRISPR and recombineering can increase the rate of mutation close to 100% due to the lethality of the DSB. Functionally, this makes CRISPR–Cas a potent and targetable counter-selectable marker [166]. The combination of recombineering and CRISPR has been used in many proteobacteria, but it can take substantial effort to transfer to organisms where transformation efficiency is low and recombineering tools do not yet exist.

The nuclease spCas9 is most commonly used for genome editing across many types of bacteria. However, Cas9 expression can be toxic in a variety of hosts [159, 167]. Toxicity can be overcome by placing the gene under an inducible promoter so that it is only expressed when needed [168], but this is only possible in organisms where inducible promoters have been developed. Toxicity can also be overcome in some organisms by mutating one of the active sites, resulting in Cas9n, so that it can only nick one strand of DNA rather than creating a DSB [169, 170]. Another issue with the commonly used Cas9 enzymes is that they are unable to function in thermophiles; therefore, thermophilic Cas9 enzymes have been identified and implemented along with thermophilic recombineering machinery for engineering thermophiles [130]. Although spCas9 is the most widely used nuclease, other CRISPR–Cas systems have been identified to overcome problems with spCas9. These nucleases also have different cleavage patterns and target different PAM sequences. Cpf1, a member of the Cas12a group, has been used in several organisms for genome editing, including those where spCas9 did not function [159, 171, 172]. Native CRISPR systems have also been leveraged for genome editing [173, 174]. There is significant interest in CRISPR based tools for non-model organisms, and it has been used wildly and in more depth elsewhere [161, 162, 175, 176].

### CRISPR/Cas for gene regulation

While CRISPR–Cas9 has been widely demonstrated for DNA insertions and deletions, it can also be used for gene regulation by silencing or activating transcription using a catalytically inactive Cas9 (dCas9). In this case, the dCas9 is unable to act as a nuclease but is still able to bind DNA at a targeted sequence. In CRISPR interference (CRISPRi), a gRNA-targeted dCas9 binds to either the promoter of the desired gene or the open reading frame (ORF), therefore blocking either transcription initiation or elongation and inhibiting gene expression (177). This enables gene knockdowns in a regulated and reversible way, and expression of multiple gRNAs can allow multiplexing of gene knockdowns [178]. Researchers have developed CRISPRi tools in a wide variety of non-model microorganisms to both study the activity of essential genes and to knockdown expression of competing pathways to increase production of fuels and chemicals [179–182].

While CRISPR can be a powerful genome engineering and gene regulation tool, it is often difficult to optimize in non-model bacteria. Therefore, research has gone into developing a modular CRISPRi system that can be deployed across phylogenetically diverse bacteria. Mobile-CRISPRi has demonstrated the gene knockdown efficacy in a diverse set of pathogenic microbes, though this system has yet to be used in organisms of interest for industrial use [183]. While CRISPRi represses transcription, dCas9 can also be used to activate transcription via CRISPR activation (CRISPRa). In CRISPRa the dCas9 is fused to a transcriptional activator so that when dCas9 binds upstream of a gene it recruits RNA polymerase leading to increased transcription. While CRISPRa has been used in *E. coli* using both PolII [184] and SoxS [185], this tool is still in the early stages of being applied to other prokaryotes [186].

## Gene expression tools in non-model microorganisms

The ability to reliably fine-tune gene expression is an important synthetic biology tool and can play an important role in increasing biofuel and chemical yields from engineered pathways. Transcription and translation levels can be controlled by several different components including promoters, riboswitches, ribosome binding sites (RBS), and terminators. Genes can be constitutively expressed, where protein production levels are largely determined by the strengths of the promoter and RBS, or genes can be regulated where expression can be turned “on” or “off” by inducible promoters and riboswitches. Reporter genes can provide an easily assayed output to help determine the impact of different genetic parts on expression levels, and they also having applications in the development of biosensors for the detection and control metabolite levels during bioconversion. Developing these gene expression tools for non-model microbes is critical to enabling rational metabolic pathway optimization for improved product formation.

### Constitutive gene expression

The simplest way to modulate gene transcription is through the evaluation of promoters with varying activity levels. Often, strong characterized promoters from model organisms, like P_tac_ from *E. coli*, are used but they do not always confer robust gene expression in non-native hosts. Identifying and implementing native promoters can overcome this issue. Strong promoters often drive expression of genes in central metabolism and identifying those genes in a target organism may help in identifying useful promoters. RNAseq can be employed on cells grown under desired culture conditions [187–189], where genes with high transcript levels may be indicative of a strong promoter. Further characterization of the identified promoters through a time course study under various growth conditions may identify those that are constitutive [189].

Overexpression with strong promoters is not always desirable, especially if an intermediate in the pathway is toxic or if a protein is membrane-bound. Furthermore, when many genes need to be expressed, the metabolic burden of protein production can become substantial [190]. Therefore, the development of a promoter library with a range of promoter strengths is also useful. Typically, the closer the − 35 and − 10 sequences are to consensus, the stronger the promoter is. Mutating each of the bases in the sequence and varying the space between them has been shown to vary promoter strength [191, 192]. Mutated promoters can also be obtained through error-prone PCR or through site-directed mutagenesis [188]. Promoter libraries have been characterized in a wide variety of non-model organisms [193–196]. Other regions that impact gene expression and can be varied include the UP element and the ribosome binding site (RBS). UP elements are sequences upstream of the − 35 box in the promoter that bind to the alpha subunit of the RNA polymerase, greatly increasing expression levels [197]. While underexplored for many non-model microbes, UP elements can bring an additional boost to expression when very high transcription levels are desired [198–200]. The RBS recruits the ribosome and it is an important control point for translation initiation. The RBS is commonly used to tune gene expression through alterations of the sequence and the space between the sequence and the start codon. Libraries of RBSs have been created in combination with promoters to fine-tune gene expression for fuel and chemical production [195, 201, 202]. An RBS calculator has been created to aid in the creation of RBS libraries and to predict translation initiation rates [203].

### Regulated gene expression

The ability to regulate gene expression is an important tool for both strain engineering and bioproduction. Applications include expression of a toxic gene (e.g., Cas9) for a short period [168, 204], balancing of metabolic flux with a biosensor [205], or turning on a production pathway once cell growth has reached stationary phase to reduce the metabolic burden [206–208]. They are also important to determine whether a gene is essential under the conditions being tested. One mechanism of creating inducible promoters involves the use of transcription factors. Well-characterized promoters from *E. coli* such as lactose- and arabinose-inducible promoters have been transferred, sometimes with mutations to enable functionality, to a wide variety of other organisms, including species of *Clostridium*, *Pseudomonas*, *Bacillus*, and *Ralstonia* [79, 209]. Other inducers have also been commonly used such as tetracycline [210], xylose [211], and nisin [94]. Newer promoters such as the Jungle Express expression system for proteobacteria [212], and less commonly used ones such as laminaribiose [213] also have the potential to be useful for engineering various non-model organisms. However, transferring well characterized promoters into non-model bacteria can still be challenging. Many result in “leaky” expression or lack regulatory proteins, such as repressors or activators, not present in heterologous hosts. In addition, even if regulatory proteins are transferred with the promoter, transport of the inducer molecule may be an issue.

Gene expression can also be regulated post-transcriptionally. Riboswitches are regulatory mRNA elements in the 5′ untranslated region that are capable of regulating gene expression through small molecule-induced structural switching [214]. They add a layer of regulation, but are less explored for the engineering of non-model microbes relative to model organisms like *E. coli*. In this mechanism, mRNA forms two distinct shapes depending on the presence of a specific small molecule, which either blocks or allows translation. Examples of the use of riboswitches for engineering of non-model microbes include theophylline for inducible production of biofuels and other compounds [215], lysine to balance flux for competing pathways [214, 216], and flavonoids such as naringenin for increased production of flavonoids [217]. Recently, thermophilic riboswitches have also been identified and used to regulate biofuel production [218]. Riboswitches are particularly beneficial for regulating gene expression in non-model microbes because they function independently of host-associated proteins and without the need for organism-specific promoters. For example, a set of theophylline riboswitches have been demonstrated to function in a many species including multiple cyanobacteria, *Mycobacterium*, and *Streptomyces*. These switches have also proven to be superior for regulation of gene expression when compared to traditional IPTG-inducible promoters [215].

### Terminators

Transcriptional terminators in *E. coli* have been well characterized and are an important tool for gene expression. Terminators can impact mRNA stability and expression levels of adjacent genes [219] Simple terminators that do not require additional termination factors have been used widely in diverse genera. Some terminators, like those from bacteriophage T7, can be inefficient at termination, causing read through on multigene constructs. However, several commonly used terminators contain repeats that increase the stability of transcriptional pausing and increase reliability, though this can cause issues when using the same terminator repeatedly use within a single construct [173]. Efficient and reliable control of multigene constructs for metabolic engineering of bacteria therefore requires several well characterized terminators. Libraries [174] and terminator prediction programs [175] have been developed, but terminator research in non-model bacteria lags behind other parts for gene expression like promoters.

### Reporter genes

Reporter genes encode proteins that can be tracked or quantified, often visually or spectrophotometrically. They have many applications in flow cytometry, microscopy, protein localization, and microbial co-cultures, and they are particularly useful when screening promoter and gene expression libraries. Enzymes can act as reporter genes, including common ones such as *lacZ* (β-galactosidase) and *uidA/gusA* (β-glucuronidase), where a substrate (e.g., X-gal) is cleaved to generate a colored compound, and enzyme activity is proportional to enzyme abundance. Other enzymes include chloramphenicol acetyltransferase (*catP*) [220] and alcohol dehydrogenase (*adhE*) [221, 222]. Determining enzymatic activity can be laborious, which makes these enzymatic reporter genes less useful, and they are typically used only as end-point assays [223].

Genes encoding fluorescent proteins are the most commonly used type of reporter gene because they can provide real-time measurements of gene expression. In aerobic microbes, several different genes encoding fluorescent protein have been engineered, including the green fluorescent protein, *gfp*, and the red fluorescent protein mCherry. Each of the fluorescent proteins has different characteristics from color to brightness to stability [224]. These genes have been used in a wide variety of applications. The fluorescent genes are used in transcriptional fusions to indicate the strength of promoters and expression vectors during bacterial cell growth [191, 225]. The simplicity and non-invasive nature of fluorescent proteins allow easier tracking of the dynamics of gene expression and allow much higher throughput. This higher throughput enables the use of these genes as biosensors by fusing the fluorescent reporter to regulated promoters that respond to the presence of metabolites, such as the target product molecule, metabolic intermediate, or toxic compound [226–228]. One caveat is that reporters are only a proxy for gene expression, and the expression level of heterologous genes can depend on the exact genetic context, especially if mRNA secondary structure or stability changes. These fluorescent reporter genes are incredibly useful in aerobic bacteria because they require O_2_ to form the fluorescent chromophore.

Many biotechnology-relevant organisms are strict anaerobes, where O_2_-dependent reporters can be more challenging to use. The use of fluorescent proteins requires that anaerobic cultures be brought into an aerobic environment, which can be lethal, and the cells often need to be washed in a time-consuming process. Therefore, they cannot be used in real-time growth experiments anaerobically. Flavin-binding fluorescent proteins (FbFPs) do not require O_2_ for fluorescence, making them potentially useful tools for anaerobes. Examples of FbFPs include iLOV, BsFbFP, PpFbFP, and EcFbFP which have been demonstrated in a variety of organisms including many species of clostridia [229]. However, FbFPs are not as bright as aerobic fluorescent genes, which make them more difficult to quantify. To overcome the limitation of FbFPs, a fluorescence-activating and absorption-shifting tag (FAST) protein has been developed. FAST relies on the presence of an exogenously added ligand that is only fluorescent when bound to FAST. Two color signals have been developed, a green yellow (YFAST) signal where 4-hydroxy-3-methylbenzylidine-rhodanine (HMBR) binds FAST and a red signal (rFAST) where 4-hydroxy-3,5-dimethoxybenzylidene-rhodanine (HBR3,5DOM) binds. FAST produces a fluorescence signal similar to that of GFP and has recently been used in a few organisms [230, 231]. This tool has the potential to revolutionize the use of reporter genes in anaerobic microbes. This includes the ability to monitor real-time gene expression, screen promoter libraries, study protein localization, and develop other high-throughput tools such as biosensors.

## Conclusion

We are entering an era of both rational and systematic design of genetic tools for the metabolic engineering of diverse non-model bacteria. Transformation methods that overcome native RM systems via targeted DNA methylation and the utilization of libraries of genetic parts is enabling the manipulation of numerous new hosts to harness their native complex phenotypes. Here we outline an approach to “domesticate” non-model organisms by rapidly developing a genetic toolbox to enable both rational and untargeted metabolic engineering. As more bacteria become genetically tractable and more tools are established in these hosts, advanced genome engineering tools like CRISPR–Cas, biosensors, and phage recombinases will further accelerate metabolic engineering efforts. The ability to fine-tune metabolic pathways in organisms that have beneficial, complex phenotypes will enable engineering for increased titer, rate, and yield for a range of important products to build a biobased, sustainable future.

## Data Availability

Not applicable.
